# Dihydromyricetin Ameliorates Exercise-Induced Acute Hepatic Dysfunction and Inflammation in Association with the Restoration of the Th17/Treg Balance in Mice

**DOI:** 10.3390/ijms27219846

**Published:** 2026-11-03

**Authors:** Yong Zhang, Jing Yue, Qianyong Zhang, Long Yi

**Affiliations:** Research Center for Nutrition and Food Safety, Chongqing Key Laboratory of Nutrition and Health, Institute of Military Preventive Medicine, Army Medical University, Chongqing 400038, China

**Keywords:** dihydromyricetin, acute exercise, Th17 cells, Treg cells, hepatic inflammation

## Abstract

Exercise induces acute hepatic dysfunction and inflammation, a condition with currently limited treatment modalities. Dihydromyricetin (DHM) may protect the liver, but its role in exercise-related hepatic dysfunction and inflammation remains to be elucidated. In this work, we investigated whether DHM intervention is associated with the restoration of the T helper 17 (Th17)/regulatory T (Treg) balance in these exercise-induced hepatic disorders.Exhaustive treadmill running was performed on male C57BL/6 mice (8 weeks old, 20–22 g) to establish a mouse model of acute hepatic dysfunction and inflammation. Mice were then administered DHM (400 mg/kg/day by oral gavage for 6 consecutive days, concurrently with the exercise protocol), DHM + recombinant interleukin-17A (IL-17A) (rIL-17A; 100 ng/day, i.p., on Days 1–4 of the exercise protocol), or DHM + anti-CD25 monoclonal antibody (aCD25 mAb; 400 µg, i.p., on the second day of acclimatization and the third day of the exercise protocol). After acute exercise, livers were harvested for histopathological analysis, protein expression profiling, and immune cell population analysis. Acute exercise induced histological changes in the liver with infiltration of inflammatory cells, elevated serum aspartate aminotransferase (AST) and alanine aminotransferase (ALT), and increased hepatic Th17 cells and the Th17/Treg ratio, without significantly altering Treg cells, compared with the non-exercise group. DHM administration attenuated these changes, and this protective effect was associated with the restoration of the Th17/Treg balance. Administration of rIL-17A or aCD25 mAb blunted the protective effect of DHM. Our results suggest that DHM administration may serve as a potential protective strategy against exercise-induced transient hepatic dysfunction and inflammation, in association with the restoration of the Th17/Treg balance in mice.

## 1. Introduction

Exercise has many health benefits and can prevent several metabolic diseases, such as obesity, type II diabetes, and cardiovascular disease [1]. Moderate exercise intensity (3.0–5.9 METs, metabolic equivalents) induces positive changes in skeletal muscle and the cardiovascular system, as well as overall well-being. On the other hand, excessive exercise stress, including a single bout of exhaustive exercise, can be deleterious and may cause organ injury, particularly in the liver [2,3,4,5]. In a study conducted by Liu et al., chronic overtraining was found to be associated with progression of hepatic fibrosis [6]. Exercise-induced hepatic dysfunction is a common exercise-associated problem, characterized by an excessive inflammatory response, oxidative stress, ischemia–reperfusion injury, and hepatocyte apoptosis [6,7,8]. Recent evidence suggests that dysregulation between T helper 17 (Th17) and FOXP3+ regulatory T (Treg) cells contributes to the induction of hepatic inflammation [9]. IL-17 has been shown to participate in various pathological processes associated with diverse clinical conditions [9,10,11,12].

Th17 cells have been implicated in the pathogenesis of acute liver injury, nonalcoholic steatohepatitis (NASH), alcohol-associated liver disease (AALD), liver fibrosis, and hepatocellular carcinoma (HCC) [13,14]. These cells produce IL-17, which, under physiological conditions, protects against bacterial and fungal pathogens [15]. IL-17A, the most abundant member of the IL-17 cytokine family, is primarily produced by CD4_+_ Th17 cells [15,16,17].

Studies have shown that Th17 cells are involved in hepatic inflammation [18], whereas Tregs exert immune-modulating effects via CD4^+^CD25^+^ cells [10,11]. Immunological dysfunction, characterized by an elevated Th17 response and/or a reduced Treg response, has been reported in many hepatic diseases [10,11]. However, whether Th17/Treg imbalance is involved in acute exercise-induced hepatic dysfunction has not been reported before. Therefore, we hypothesize that a shift in the balance between Th17 and Tregs could play an important role in the pathogenesis of exercise-associated hepatic damage.

DHM, also known as ampelopsin, is a natural flavonoid isolated from *Ampelopsis grossedentata.* In mice, it has been shown to ameliorate high-fat diet (HFD)-induced hepatic steatosis, alleviate inflammation, and prevent the progression of nonalcoholic fatty liver disease (NAFLD). Additionally, research shows that DHM ameliorates the pathologies of NASH, including fatty liver disease, inflammation, and fibrosis, potentially via the regulation of the SIRT1 signaling pathway [19,20]. However, how DHM ameliorates hepatic inflammation still needs further investigation to explore its specific mechanism.

Interestingly, certain plant compounds have been shown to exert immunoregulatory effects on the Th17/Treg balance [21,22]. Therefore, we investigated whether DHM ameliorates exercise-induced hepatic dysfunction and whether this effect is associated with the Th17/Treg balance.

## 2. Results

### 2.1. Acute Exercise Induces Hepatic Dysfunction and Inflammation and Disrupts the Hepatic Th17/Treg Balance

**Figure 1:**
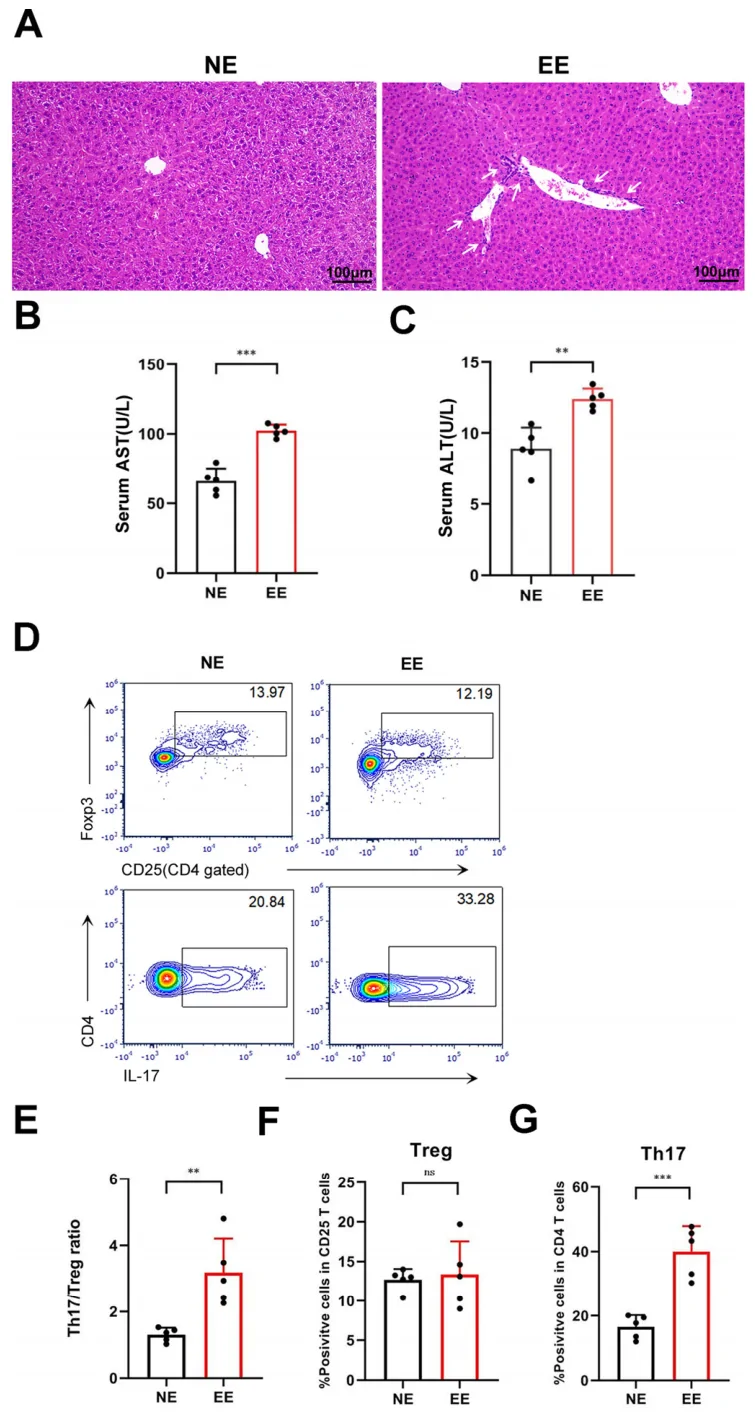
Acute exercise induces hepatic dysfunction and inflammation and disrupts hepatic Th17/Treg balance. (**A**) Representative images of H&E staining of liver sections (*n* = 5 per group, original magnification: 100×). Hepatic sinusoid aggregation of inflammatory cells was observed in the EE group, while liver tissues in the NE group appeared normal. Arrows indicate inflammatory cell infiltration and sinusoidal congestion. Scale bars: 100 µm. (**B**,**C**) Serum levels of AST (**B**) and ALT (**C**) in mice (*n* = 5 per group). (**D**) Representative flow cytometry plots of Foxp3 and IL-17 expression in gated intrahepatic CD4^+^ T cells from NE and EE mice. (**E**–**G**) Quantification of the percentages of Th17/Treg (**E**), Treg (**F**), and Th17 (**G**) cells in (**D**) (*n* = 5 per group). Data are presented as means ± SD. Normality was assessed using the Shapiro–Wilk test, and homogeneity of variance was assessed using the Brown-Forsythe and Bartlett’s tests. Given the relatively small sample sizes, parametric tests (Student’s *t*-test or Welch’s *t*-test when variances were unequal) were applied when the data were approximately normally distributed, with Brown-Forsythe used as the primary criterion when the two variance tests yielded conflicting results. A *p* value < 0.05 was considered statistically significant. ** *p* < 0.01, *** *p* < 0.001; ns, not significant. NE, non-exercise group; EE, exhaustive exercise group; H&E, hematoxylin and eosin; AST, aspartate aminotransferase; ALT, alanine aminotransferase; Th17, T helper 17 cell; Treg, regulatory T cell; Foxp3, forkhead box P3; IL-17, interleukin-17.

The effects of exhaustive exercise on hepatic histology were assessed by examining morphological changes in the liver tissue of mice, stained with hematoxylin and eosin (H&E). The non-exercise (NE) liver tissue showed an intact architecture with intact sinusoidal spaces and normal hepatocytes. In contrast, exhaustive exercise (EE) induced acute hepatic dysfunction and inflammation, characterized by sinusoidal congestion and inflammatory cell infiltration (Figure 1A). In this study, EE increased serum levels of biochemical markers of hepatocellular alteration, including AST and ALT, compared with the NE group (Figure 1B,C). Next, we investigated whether EE was associated with the balance between Th17 and Treg cells. As shown in Figure 1D–G, we observed an increase in Th17 cells in the liver tissues of EE mice compared with NE mice, and the Th17/Treg ratio was increased (Figure 1E,G).

### 2.2. DHM Ameliorates Exercise-Induced Acute Hepatic Dysfunction and Inflammation

**Figure 2:**
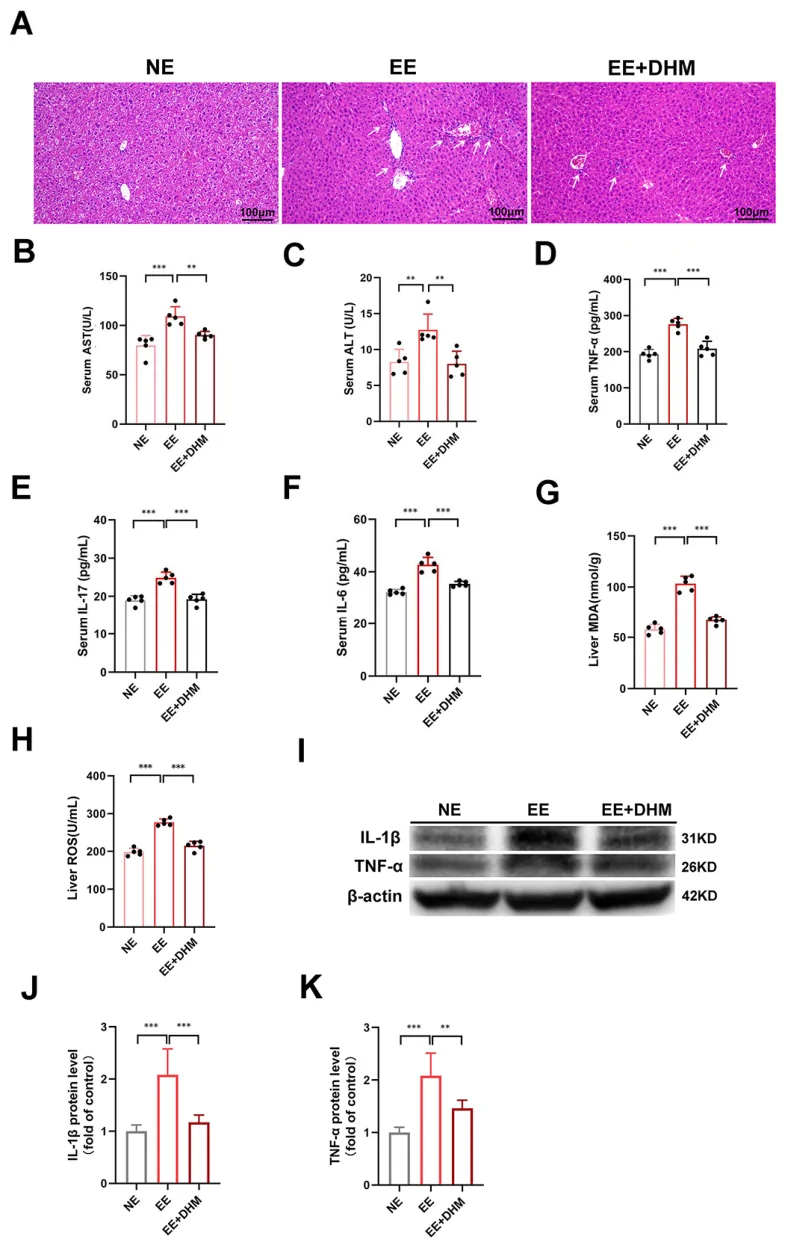
DHM ameliorates exercise-induced acute hepatic dysfunction and inflammation. (**A**) Representative images of H&E staining of liver sections (*n* = 5 per group, original magnification: 100×). Hepatic sinusoid aggregation of inflammatory cells was observed in the EE group, while liver tissue inflammation was reduced in the EE+DHM group. Arrows indicate inflammatory cell infiltration and sinusoidal congestion. Scale bars: 100 µm. (**B**,**C**) Serum levels of AST (**B**) and ALT (**C**) in mice (*n* = 5 per group). (**D**–**F**) Serum levels of inflammatory cytokines in mice (*n* = 5 per group). (**G**,**H**) Hepatic levels of oxidative stress markers in mice (*n* = 5 per group). (**I**–**K**) Representative Western blots and quantified protein levels of IL-1β (**J**) and TNF-α (**K**) in liver tissue (*n* = 5 per group). Data are presented as means ± SD. Normality was assessed using the Shapiro–Wilk test, and homogeneity of variance was assessed using the Brown–Forsythe and Bartlett’s tests. Given the relatively small sample sizes, parametric tests (one-way ANOVA followed by Tukey’s post hoc test) were applied when the data were approximately normally distributed, with Brown-Forsythe used as the primary criterion when the two variance tests yielded conflicting results. A *p* value < 0.05 was considered statistically significant. ** *p* < 0.01, *** *p* < 0.001. NE, non–exercise group; EE, exhaustive exercise group; EE + DHM, exhaustive exercise with dihydromyricetin treatment; H&E, hematoxylin and eosin; AST, aspartate aminotransferase; ALT, alanine aminotransferase; ROS, reactive oxygen species; MDA, malondialdehyde; IL-1β, interleukin-1 beta; TNF-α, tumor necrosis factor alpha.

Liver tissues from the NE group exhibited intact architecture with normal hepatocytes and sinusoidal spaces. In contrast, EE induced acute hepatic dysfunction and inflammation, characterized by sinusoidal congestion and inflammatory cell infiltration. DHM administration reduced these pathological changes, although mild sinusoidal congestion was still observed. The histological findings for the NE, EE, and EE + DHM groups are presented in Figure 2A.

Exhaustive exercise increased serum levels of AST and ALT, biochemical markers of hepatocellular alteration, compared with the NE group. DHM administration attenuated these elevations compared with the EE group (Figure 2B,C).

ELISA analysis revealed that EE increased the levels of proinflammatory cytokines, including TNF-α, IL-17, and IL-6, in serum. Concurrent administration of DHM reduced the levels of these cytokines compared with the EE group (Figure 2D–F).

To investigate the antioxidant potential of DHM in EE-induced acute hepatic dysfunction, we measured oxidative stress markers, including ROS and MDA, in the liver tissue. EE increased the levels of ROS and MDA compared with the NE group. DHM administration reduced these levels compared with the EE group (Figure 2G,H).

Consistent with histopathological observations, EE enhanced the expression of pro-inflammatory cytokines IL-1β and TNF-α at the protein level within the liver tissue compared with the NE group. In contrast, DHM administration attenuated these effects (Figure 2I–K).

### 2.3. DHM Effects Are Associated with the Hepatic Th17/Treg Balance

**Figure 3:**
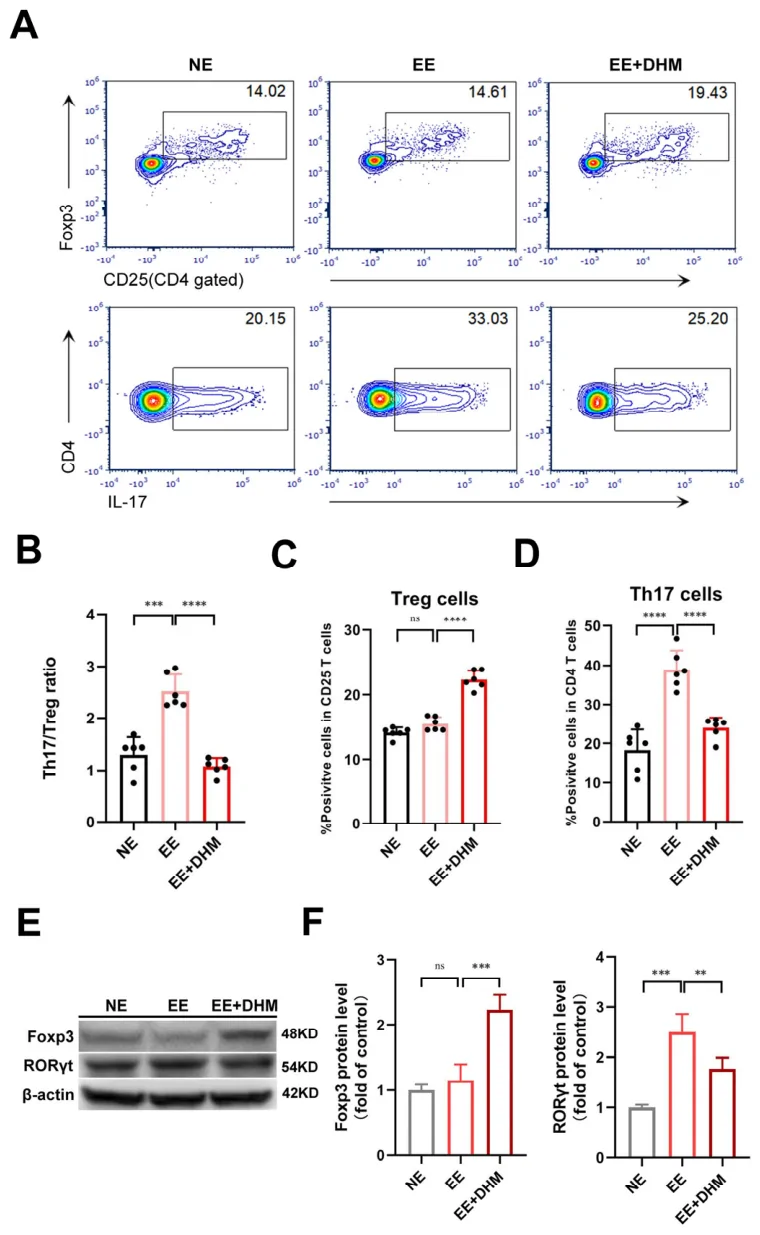
DHM effects are associated with the hepatic Th17/Treg balance. (**A**) Representative flow cytometry plots of Foxp3 and IL-17 expression in gated intrahepatic CD4^+^ T cells from the liver of NE, EE, and EE+DHM mice. (**B**–**D**) Quantification of the percentages of Th17/Treg (**B**), Treg (**C**), and Th17 (**D**) cells in (**A**) (*n* = 6 per group). (**E**,**F**) Immunoblot analysis of Foxp3 and RORγt in the liver of NE, EE, and EE+DHM mice; β-actin was used as an internal control (*n* = 5 per group). Data are presented as means ± SD. Normality was assessed using the Shapiro–Wilk test, and homogeneity of variance was assessed using the Brown–Forsythe and Bartlett’s tests. Given the relatively small sample sizes, parametric tests (one-way ANOVA followed by Tukey’s post hoc test) were applied when the data were approximately normally distributed, with Brown–Forsythe used as the primary criterion when the two variance tests yielded conflicting results. A *p* value < 0.05 was considered statistically significant. ** *p* < 0.01, *** *p* < 0.001,**** *p* < 0.0001; ns, not significant. NE, non-exercise group; EE, exhaustive exercise group; EE + DHM, exhaustive exercise with dihydromyricetin treatment; Th17, T helper 17 cell; Treg, regulatory T cell; Foxp3, forkhead box P3; RORγt, retinoic acid-related orphan receptor gamma t; IL-17, interleukin-17.

Next, we investigated whether the beneficial effects of DHM administration were associated with the balance between Th17 and Treg cells. As shown in Figure 3A–D, EE increased the Th17/Treg ratio and the proportion of Th17 cells among CD4^+^ T cells, but did not alter the proportion of Treg cells among CD4^+^ T cells compared with the NE group. DHM administration reduced the proportion of Th17 cells among CD4^+^ T cells and increased the proportion of Treg cells among CD4^+^ T cells in the liver compared with the EE group, resulting in a decrease in the Th17/Treg ratio. Additionally, a similar inverse expression pattern was observed for the key transcription factors Foxp3 and RORγt (Figure 3E,F): EE did not alter Foxp3 expression but increased RORγt expression compared with the NE group. To further support the flow cytometric findings, we also examined Foxp3 and RORγt protein levels in whole liver lysates. DHM administration increased Foxp3 expression and decreased RORγt expression in whole liver lysates, consistent with the flow cytometric findings of increased Treg and decreased Th17 frequencies.

### 2.4. DHM-Mediated Protective Effects Are Associated with the Th17/Treg Balance

**Figure 4:**
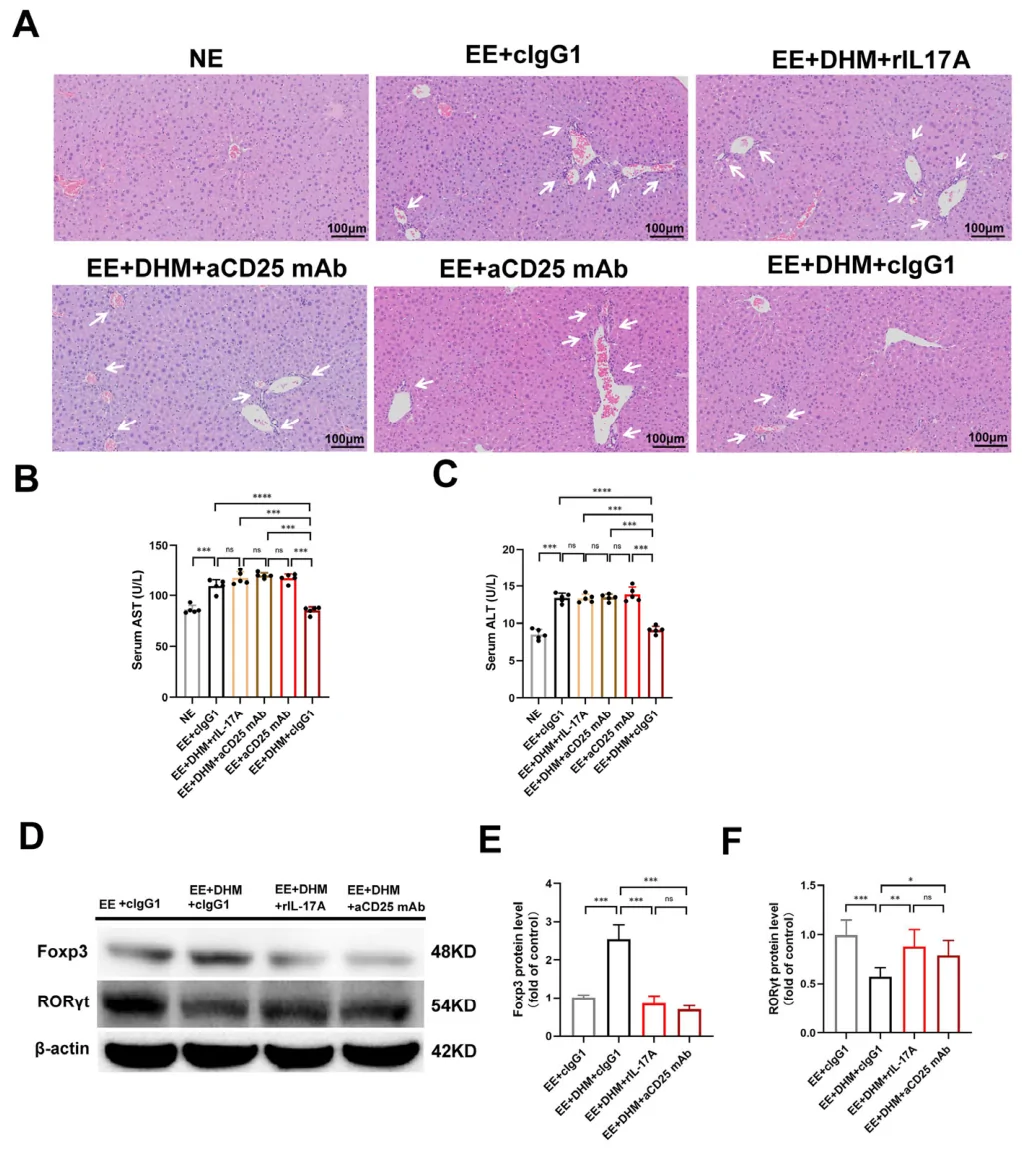
DHM-mediated protective effects are associated with the Th17/Treg balance. (**A**) Representative images of H&E staining of liver sections from NE, EE+cIgG1, EE+DHM+rIL-17A, EE+DHM+aCD25 mAb, EE+aCD25 mAb, and EE+DHM+cIgG1 groups (*n* = 5 per group, original magnification: 100×). Hepatic sinusoid aggregation of inflammatory cells was observed in the EE+cIgG1, EE+DHM+rIL-17A, EE+DHM+aCD25 mAb, and EE+aCD25 mAb groups, while liver tissues in the EE + DHM + cIgG1 group appeared to improve. Arrows indicate inflammatory cell infiltration and sinusoidal congestion. Scale bars: 100 µm. (**B**,**C**) Serum levels of AST (**B**) and ALT (**C**) in mice (*n* = 5 per group). (**D**–**F**) Representative Western blots and quantified protein levels of Foxp3 and RORγt in liver tissue (*n* = 5 per group); β-actin was used as loading control. Data are presented as means ± SD. Normality was assessed using the Shapiro–Wilk test, and homogeneity of variance was assessed using the Brown–Forsythe and Bartlett’s tests. Given the relatively small sample sizes, parametric tests (one-way ANOVA followed by Tukey’s post hoc test) were applied when the data were approximately normally distributed, with Brown–Forsythe used as the primary criterion when the two variance tests yielded conflicting results. A *p* value < 0.05 was considered statistically significant. * *p* < 0.05, ** *p* < 0.01, *** *p* < 0.001,**** *p* < 0.0001; ns, not significant. NE, non-exercise group; EE, exhaustive exercise group; EE+DHM, exhaustive exercise with dihydromyricetin treatment; cIgG1, control IgG1; rIL-17A, recombinant interleukin-17A; aCD25 mAb, anti-CD25 monoclonal antibody; H&E, hematoxylin and eosin; AST, aspartate aminotransferase; ALT, alanine aminotransferase; Foxp3, forkhead box P3; RORγt, retinoic acid-related orphan receptor gamma t.

Considering that DHM could restore the Th17/Treg balance, we hypothesized that the protective effects of DHM are associated with this balance. To evaluate the effects of DHM-induced reduction in Th17 cells in EE-induced hepatic dysfunction and inflammation, rIL-17A was administered to mice via intraperitoneal injection on Days 1–4 of the exercise protocol. Administration of rIL-17A was associated with a loss of the protective effect of DHM in mice with EE-induced hepatic dysfunction and inflammation (Figure 4A–C). Next, to investigate the role of Treg cells, an anti-CD25 monoclonal antibody (aCD25 mAb), which partially depletes Treg cells, was administered on the second day of acclimatization and the third day of the exercise protocol. Similarly, intraperitoneal injection of aCD25 mAb was associated with a loss of the protective effect of DHM in mice with EE-induced hepatic dysfunction and inflammation (Figure 4A–C). The reversal of DHM administration was comparable between rIL-17A and aCD25 mAb treatments (Figure 4A–C). The results of the Western blot analysis were also consistent with the aforementioned findings (Figure 4D–F). Taken together, these results suggest that the protective effects of DHM are associated with the restoration of the Th17/Treg balance.

## 3. Discussion

Although moderate-intensity exercise benefits the liver, excessive training may harm the organ. One possibility is that reduced perfusion in visceral tissue during intense training, combined with oxidative injury, leads to the release of bacterial toxins, activation of Kupffer cells, and hepatic inflammation [3,10]. Muscle-derived lactate vesicles have also been reported to induce hepatic apoptosis and stellate cell activation via the FBXO2/MCL1/BAX pathway, linking muscular energy depletion to liver fibrosis [6]. Our findings indicate that the Th17/Treg balance is also disturbed in exercise-induced hepatic dysfunction and inflammation, which may interact with these known mechanisms and contribute to the transition from acute injury to chronic disease.

Taken together, our results show that DHM administration ameliorates EE-induced hepatic dysfunction and inflammation in association with the restoration of the Th17/Treg balance, as evidenced by increased intrahepatic Treg cells and decreased Th17 cells. Recent studies have shown that several flavonoids, including DHM, are hepatoprotective in a wide range of hepatic injuries via mechanisms such as enhanced antioxidant activity and inhibition of CYP2E1 and NF-κB signaling [23,24,25]. In this study, we describe a potential anti-inflammatory effect of DHM, in line with recent publications that emphasize the importance of Treg induction in counteracting inflammation-induced injury during chronic liver diseases [26,27].

The Th17/Treg ratio is relevant for the regulation of immunity, particularly when the liver is damaged. Tregs expressing Foxp3 are essential for maintaining immune homeostasis and preventing autoimmunity and excessive inflammation [28,29], whereas IL-17-producing Th17 lymphocytes are pro-inflammatory cells involved in a wide range of inflammatory and autoimmune diseases [30,31]. Indeed, exogenous IL-17A has been reported to exacerbate hepatic ischemia–reperfusion injury in fatty liver by promoting neutrophil infiltration and mitochondria-driven apoptosis, underscoring the pathogenic role of IL-17A in hepatic injury [32]. Our results showed that DHM administration increased Foxp3 expression and enhanced hepatic Tregs, while Th17 differentiation was reduced. This suggests that DHM may modulate immune function to reduce hepatic dysfunction and inflammation. Tregs exert their suppressive function via cytokines such as IL-10 and TGF-β [33,34,35]. IL-10 has broad immunosuppressive properties and downregulates pro-inflammatory cytokines such as TNF-α and IL-6, which are generally overexpressed in hepatic injury [36,37,38]. TGF-β suppresses effector T cell function while converting naïve T cells into iTregs [39,40]. However, IL-10 and TGF-β were not directly measured in the present study, and their potential involvement in the observed effects remains to be determined. The balance between Treg cells and Th17 cells depends on the local cytokine environment: low TGF-β levels together with IL-6 promote Th17 differentiation, whereas high TGF-β levels alone promote iTreg generation [41,42]. This equilibrium must be maintained for an appropriate immune response; otherwise, excessive Th17 cells may cause chronic inflammation and tissue injury [43]. ROS are also important signaling messengers that influence CD4^+^ T cell fate. Low levels of ROS generally favor Treg differentiation, whereas excessive ROS, together with pro-inflammatory cytokines such as IL-6 and TGF-β, promotes Th17 differentiation. In the present study, exhaustive exercise increased hepatic ROS and MDA levels and was associated with an increased Th17/Treg ratio, whereas DHM treatment reduced ROS and MDA levels and was associated with the restoration of the Th17/Treg balance. These findings suggest that redox status may contribute to the regulation of the Th17/Treg balance, although the precise ROS and signaling pathways involved were not examined. Future studies using ROS-specific probes and ROS scavengers will help to clarify this link. Our study suggests that DHM may shift this balance toward regulatory T cells in association with Foxp3 upregulation, thereby reducing Th17-mediated inflammation.

This is consistent with other studies showing that Tregs reduce inflammation in liver diseases by inhibiting HSC activation and fibrotic progression [44] and by downregulating pro-inflammatory cytokines such as TNF-α and IL-6 [45,46]. The protective effect of DHM against EE-induced hepatic dysfunction and inflammation is particularly significant because it is associated with the modulation of the Th17/Treg balance and inflammatory cytokines in the present study. This is consistent with previous reports showing that DHM can also regulate macrophage polarization [47] and ameliorate hepatic injury through multiple mechanisms [48]. This is particularly important given the increasing prevalence of hepatic disorders such as ALD and NAFLD [49,50]. The dose of 400 mg/kg/day was selected based on previously published preclinical studies demonstrating hepatoprotective effects of DHM in rodent models [51]. This relatively high dose was used for two reasons. First, the oral bioavailability of DHM is relatively low, and the systemic exposure achieved at 400 mg/kg in mice may not be directly comparable to that in humans [52]. Second, DHM was administered for only 6 consecutive days in the present study, whereas human trials have typically used longer intervention periods; a higher daily dose over a short period was therefore used to observe a measurable short-term effect. In our randomized controlled trial in patients with nonalcoholic fatty liver disease, DHM was administered orally at 600 mg/day (two 150 mg capsules twice daily) for 3 months, corresponding to approximately 8–10 mg/kg/day in a 60 kg adult, which is substantially lower than the 400 mg/kg/day used in the present study [53]. Therefore, the dose used in the present study is not directly translatable to human supplementation, and further dose-optimization studies are needed. No obvious signs of toxicity were observed in DHM-treated mice during the treatment period, although a comprehensive toxicity assessment was not performed in the present study [54]. Overall, natural DHM may serve as a potential protective strategy for liver diseases [55], as it has been reported to regulate immune homeostasis [56] and to reduce IL-17A and Th17 populations via the RORγt pathway [57]. Future studies should consider the synergistic effects of DHM with antioxidants or anti-inflammatory agents [58], and further preclinical and clinical investigations are warranted [53,59,60].

Several limitations should be acknowledged. First, rIL-17A was administered on Days 1–4 of the exercise protocol; given the short half-life of IL-17A, the direct effect of exogenous rIL-17A at the time of exhaustive exercise may be limited. This does not exclude the possibility that rIL-17A acted during the early phase of the exercise protocol to modulate the hepatic immune microenvironment, which would be consistent with the observed blunting of DHM’s protective effect. Second, Treg depletion efficiency was not directly assessed by flow cytometry. Although PC61 has been widely used for Treg depletion, its efficiency varies across tissues and models [61,62]; therefore, the contribution of Tregs may be underestimated rather than overestimated in our study. Third, it should be noted that the cellular source of IL-17A was not comprehensively examined in the present study. Although CD4^+^ Th17 cells are a major source of IL-17A, other cell types, including γδ T cells, CD8^+^ T cells, NKT cells, and innate lymphoid cells, can also produce IL-17A and may contribute to the observed inflammatory response. Future studies examining IL-17A-producing cell subsets in the liver, including γδ T cells, CD8^+^ T cells, NKT cells, and innate lymphoid cells, will help to clarify the cellular sources of IL-17A in exercise-induced hepatic inflammation. Fourth, the histological assessment was qualitative and descriptive only. No quantitative histological scoring was performed, and the severity of hepatic histopathological changes should therefore be interpreted with caution. Fifth, the composition of the inflammatory cell infiltrate was not validated with specific markers such as Ly6G, MPO, CD3, CD4, CD8, F4/80, CD68, or CD11b in the present study. Therefore, the precise identity of the infiltrating immune cells should be interpreted with caution. Future studies using these markers will help to more precisely define the composition of the inflammatory cell infiltrate in exercise-induced hepatic inflammation. Sixth, the present study measured ROS and MDA as general markers of oxidative stress, but did not assess the activity or expression of specific antioxidant enzymes such as SOD, Gpx, Cat, Trx, Trx reductase, glutathione reductase, or peroxiredoxin, nor did it identify the specific ROS (e.g., superoxide, hydrogen peroxide, or hydroxyl radical) involved. Future studies measuring these antioxidant enzymes and using ROS-specific probes, scavengers, or pharmacological inhibitors will help to further clarify the antioxidant effects of DHM and the molecular mechanisms linking ROS to the Th17/Treg balance. Seventh, Foxp3 and RORγt protein levels were measured in whole liver lysates, which may be influenced by changes in cell composition and inflammatory infiltration. Future studies using qPCR or expression analysis of *Foxp3*, *Rorc*, *Il17a*, *Il10*, and *Tgfb1* in sorted CD4^+^ T cells will help to further validate the altered differentiation status at the single-cell level. Eighth, the sample size was relatively small and was based on previous studies rather than a formal a priori power calculation. Future studies with larger sample sizes and formal power calculations will help to further validate our findings. Ninth, the exercise model and gavage administration were not performed under blinded conditions, which may have introduced a potential bias; however, histological scoring, flow cytometry analysis, and Western blot quantification were performed by investigators blinded to group allocation. Tenth, the spatial distribution of Th17 and Treg cells in the liver was not assessed by immunohistochemistry or immunofluorescence in the present study. Future studies using IHC or IF approaches at high magnification will help to confirm the presence and localization of these immune cell subsets in situ and to further validate the flow cytometric findings. Eleventh, cell viability was not directly assessed by a viability dye in the flow cytometric analysis, and histological assessment of cell viability was not performed. Given the relatively high dose of DHM used in the present study, future studies incorporating viability dyes, TUNEL staining, or cleaved caspase-3 staining will help to further confirm that the observed effects are not confounded by changes in cell viability. Twelfth, the present study did not include a NE + DHM control group, so the potential baseline effects of DHM in non-exercised mice could not be evaluated. Future studies including this control group will help to further clarify the effects of DHM under baseline conditions. Thirteenth, time to exhaustion and total running distance were not recorded in this study. This limitation may affect the comparability of exercise exposure between groups, as differences in exercise tolerance could influence the magnitude of the physiological stimulus and potentially confound the interpretation of the protective effects of DHM. Furthermore, the partial depletion efficiency of anti-CD25 mAb and the absence of a non-exercised DHM control group should be considered as limitations when interpreting the mechanistic role of the Th17/Treg balance.

Consequently, the observed effects are described as associated with rather than causal, and future studies are needed to confirm the causal role of the Th17/Treg balance in the protective effect of DHM.

In conclusion, our study shows that DHM administration ameliorates EE-induced hepatic dysfunction and inflammation in association with the restoration of the Th17/Treg balance in the liver. Our findings highlight the potential of DHM, a naturally occurring flavonoid, as a protective dietary compound against EE-induced hepatic dysfunction and inflammation.

## 4. Materials and Methods

### 4.1. Animals and Experimental Procedures

Male C57BL/6 mice weighing 20–22 g at 8 weeks of age were housed under controlled temperature (22–25 °C) and humidity (50–55%) conditions. The mice were maintained on a 12 h light/dark cycle in the Experimental Animal Center of the Third Military Medical University (Chongqing, China). They were fed a standard chow diet (10% fat, 70% carbohydrate, 20% protein; D12450B, Research Diets), with food and water replaced every 3 days and provided ad libitum. A mouse model of acute hepatic dysfunction and inflammation was established by subjecting male C57BL/6 mice to exhaustive running exercise, as previously described with modifications [4]. A total of 58 mice were used throughout this study. Mice were randomly assigned to experimental groups using a computer-generated randomization sequence. Body weight and food intake were monitored daily throughout the study. The sample size (*n =* 5 or *n* = 6 per group) was determined based on previously published studies using similar mouse models of exercise-induced hepatic dysfunction and inflammation [4,6,47], in which *n* = 5–6 per group was sufficient to detect statistically significant differences in serum AST/ALT levels, hepatic cytokine levels, and Th17/Treg frequencies.

Acclimatization and exercise protocol. All mice underwent a 5-day acclimatization period on a motorized treadmill (SANS Biological Technology, Nanjing, China) at a speed of 5 m/min with a 0°slope for 10 min daily. Mice that failed to adapt to treadmill running were excluded from the study. After acclimatization, mice in the exercise groups underwent a 6-day exercise protocol. On Days 1–5, mice ran at 5 m/min for 10 min, followed by 10 m/min for 50 min. On Day 6, mice ran at 25 m/min until exhaustion, defined as the inability to run for 20 s despite continuous prodding by an electrode slice [63]. Mice in the NE group underwent the same 5-day acclimatization but were kept in cages without the formal exercise protocol. Mice were euthanized 1 h after the last exercise session on Day 6.

Experiment 1 (*n* = 10). Ten mice were randomly divided into two groups (*n* = 5 per group): NE and EE. Mice in the EE group performed the 6-day exercise protocol as described above. These mice were used for the analyses shown in Figure 1.

Experiment 2 (*n* = 18). Eighteen mice were randomly divided into three groups (*n* = 6 per group): NE, EE, and EE + DHM. DHM was administered by oral gavage once daily at a dose of 400 mg/kg body weight for 6 consecutive days (Days 1–6 of the exercise protocol) [51]. These mice were used for the analyses shown in Figure 2 and Figure 3. For the analyses shown in Figure 2, 5 randomly selected mice per group were used; for flow cytometric analysis (Figure 3), all 6 mice per group were used; for Western blot analysis in Figure 3, 5 randomly selected mice per group were used.

Experiment 3 (*n* = 30). Thirty mice were randomly divided into six groups (*n* = 5 per group): NE, EE + control IgG1 (EE + cIgG1), EE + DHM + recombinant IL-17A (EE + DHM + rIL-17A), EE + DHM + anti-CD25 monoclonal antibody (EE + DHM + aCD25 mAb), EE + aCD25 mAb, and EE + DHM + cIgG1. DHM was administered as in Experiment 2. For exogenous rIL-17A administration, mice were injected intraperitoneally (i.p.) with 100 ng of rIL-17A (Cat #210-17, Peprotech, Rocky Hill, NJ, USA) daily on Days 1-4 of the exercise protocol [64,65]. For Treg cell depletion, mice were injected i.p. with 400 µg of anti-CD25 monoclonal antibody (aCD25 mAb; Cat #102002, BioLegend, San Diego, CA, USA; clone: PC61) or control IgG1 (cIgG1; Cat #401902, BioLegend) on the second day of acclimatization (P1) and the third day of the exercise protocol (P7, i.e., 6 days after the first dose). This dual-dose regimen was adapted from a previously described protocol [61], and anti-CD25 antibody-mediated depletion of CD25^+^ cells has been shown to significantly reduce the percentage of Treg cells [62,66,67]. rIL-17A, aCD25 mAb, and cIgG1 were dissolved in phosphate-buffered saline (PBS). These mice were used for the analyses shown in Figure 4.

Following the exhaustion test on Day 6, mice were deeply anesthetized with isoflurane and euthanized via cervical dislocation 1 h after the last exercise session. Blood samples were collected via retro-orbital bleeding before euthanasia. Serum was separated by centrifugation at 3000× *g* for 10 min at 4 °C and stored at −80 °C until analysis. Serum was used for the measurement of AST, ALT, TNF-α, IL-17, and IL-6 levels, and liver tissues were used for the measurement of MDA and ROS levels. Livers were harvested for histological, biochemical, and flow cytometric analyses.

### 4.2. Ethics Statement

The animal study protocol was reviewed and approved by the Ethics Committee of the Army Medical University. Animal husbandry and care were carried out in compliance with the China Laboratory Animal Management Regulations and the Guide for the Care and Use of Laboratory Animals (Institute for Laboratory Animal Research, Washington, DC, USA).

### 4.3. Histological Analysis

Liver tissues were dissected, fixed in 4% (*w*/*v*) formaldehyde for at least 24 h, embedded in paraffin, sectioned at 5 µm, and stained with hematoxylin and eosin (H&E). Microscopic examination was performed, and images were captured using a light microscope (Nikon, Tokyo, Japan). Histological sections were examined by an investigator who was blinded to the group allocation. Histopathological analysis of H&E-stained sections revealed obvious inflammatory injury in the liver tissues. Specifically, prominent inflammatory cell infiltration was observed, which was diffusely distributed in the hepatic lobules and aggregated around the portal areas. No obvious hepatocellular necrosis or fibrous tissue proliferation was detected in the observed fields.

### 4.4. Enzyme-Linked Immunosorbent Assay (ELISA)

The concentrations of cytokines, including IL-17 (Cat #RX203067), IL-6 (Cat #RX203049), and TNF-α (Cat #RX202412M), were measured in serum using commercial ELISA kits (all purchased from RX, Quanzhou, China) according to the manufacturer’s protocols.

### 4.5. Biochemical Analysis

Serum AST and ALT levels were measured as biochemical markers of hepatocellular alteration. (ALT; Cat #RX203246M) and (AST; Cat #RX201960M) using a colorimetric spectrophotometer (SpectraMax M2, Molecular Devices, San Jose, CA, USA) according to the manufacturer’s instructions. Hepatic oxidative stress markers, including malondialdehyde (MDA) and reactive oxygen species (ROS), were also measured using a colorimetric spectrophotometer (SpectraMax M2, Molecular Devices, San Jose, CA, USA).

### 4.6. Western Blot Analysis

Liver tissues were weighed, and approximately 20 mg of tissue was homogenized in 300 µL of lysis buffer. Proteins were extracted from liver tissues using lysis buffer (Thermo Scientific, Waltham, MA, USA) with a protease inhibitor cocktail (Roche Diagnostics, Mannheim, Germany). All samples from different groups were processed in parallel on the same gel and transferred to the same membrane. Protein concentration was determined using a BCA Protein Assay Kit (Thermo Scientific), and equal amounts of total protein (40 µg per lane) were loaded. Samples were separated by sodium dodecyl sulfate-polyacrylamide gel electrophoresis (SDS-PAGE) and transferred to nitrocellulose membranes. The membranes were blocked with 5% (*w*/*v*) skim milk for 1 h and incubated with primary antibodies overnight at 4 °C. After incubation with peroxidase-conjugated secondary antibodies for 1 h at room temperature, proteins were visualized using a ChemiDoc MP Imaging System (Bio-Rad, Hercules, CA, USA) with Immobilon ECL substrate (Millipore, Billerica, MA, USA). Primary antibodies included anti-IL-1β (1:1000, Cat #26048-1-AP; Proteintech, Wuhan, China), anti-TNF-α (1:1000, Cat #17590-1-AP; Proteintech, Wuhan, China), anti-Foxp3 (1:1000, Cat #85128-6-RR; Proteintech, Wuhan, China), anti-RORγt (1:1000, Cat #DF3196; Affinity, Cincinnati, OH, USA), and β-actin (1:1000; Santa Cruz, Dallas, TX, USA). β-actin was used as a loading control, and protein levels were normalized to β-actin. Western blot images were quantified using Fiji/ImageJ software (Version 1.53c; National Institutes of Health, Bethesda, MD, USA) by an investigator who was blinded to the group allocation.

### 4.7. Flow Cytometry Analysis

Hepatic lymphocyte isolation. Liver tissues were minced and gently homogenized through a 70 µm nylon mesh in RPMI 1640 medium. The homogenate was kept on ice for 10 min to allow natural sedimentation, and the supernatant was collected and centrifuged at 696× *g* for 10 min at 4 °C. The cell pellet was washed twice with 1× PBS and resuspended in 3 mL of 44% (*v/v*) Percoll solution. This suspension was carefully layered onto 3 mL of 67% (*v/v*) Percoll solution in a new centrifuge tube. After centrifugation at 702× *g* for 20 min at 20 °C (acceleration 3, deceleration 0), a distinct white ring layer was visible at the interface between the 44% and 67% (*v/v*) Percoll layers, which contained the hepatic lymphocytes. The upper layer consisted of hepatocyte debris, and the bottom layer contained hepatocytes and red blood cells. Lymphocytes were collected from the interface, washed twice with 1× PBS, and resuspended in RPMI 1640 complete medium. No collagenase digestion or red blood cell lysis was performed in the present study; parenchymal cells and red blood cells were removed by Percoll density gradient separation.

Th17/Treg staining. For Th17 and Treg detection, 1 × 10^6^ cells per sample were resuspended in 200 µL of culture medium containing BD Leukocyte Activation Cocktail with BD GolgiPlug (containing PMA, ionomycin, and Brefeldin A; BD Biosciences, Cat. No. 550583, Milpitas, CA, USA) at 2 µL/mL and incubated at 37 °C with 5% CO_2_ for 5 h. Cells were then collected and washed, and surface staining was first performed with fluorochrome-conjugated monoclonal antibodies against CD45, CD3e, CD4, and CD25 at the manufacturers’ recommended concentrations for 30 min at 4 °C in the dark. After washing, cells were fixed, permeabilized, and subjected to intracellular/nuclear staining using the eBioscience Foxp3/Transcription Factor Staining Buffer Set (Invitrogen/Thermo Fisher Scientific, Cat. No. 00-5523-00, Waltham, MA, USA) according to the manufacturer’s instructions. Intracellular IL-17A and nuclear Foxp3-specific fluorochrome-conjugated antibodies were then added and incubated for at least 30 min at room temperature in the dark. After washing, cells were resuspended in Stain buffer and analyzed on a FACSCelesta flow cytometer (Becton Dickinson, Franklin Lakes, NJ, USA).

Antibodies and reagents. The following antibodies were used: anti-CD45 (clone 30-F11, BD; AF700), anti-CD3e (clone 145-2C11, BD; PerCP-Cy5.5), anti-CD4 (clone GK1.5, BioLegend; FITC), anti-CD25 (clone PC61, BioLegend; BV786), anti-IL-17A (clone TC11-18H10.1, BioLegend; BV421), and anti-Foxp3 (clone FJK-16s, Invitrogen/eBioscience; PE). β-Mercaptoethanol (55 µM), 20% (*v/v*) FBS, and 1% (*v/v*) penicillin-streptomycin were added to RPMI 1640 medium for cell culture.

Gating strategy. Debris and dead cells were excluded based on FSC/SSC morphology. Gating strategy and validation. A minimum of 100,000 events were acquired for each sample. The gating strategy for hepatic Th17 and Treg cells is shown in Supplementary Figure S1. Lymphocytes were first gated on FSC-A/SSC-A, followed by single cells on FSC-A/FSC-H. CD45^+^ cells were gated on Comp-Alexa Fluor 700-A versus FSC-H, and CD3^+^CD4^+^ T cells on Comp-PerCP-Cy5.5-A versus Comp-FITC-A. Th17 cells (IL-17A^+^) were gated on Comp-BV421-A versus Comp-FITC-A, and Treg cells (CD25^+^Foxp3^+^) on Comp-BV786-A versus Comp-PE-A. An FMO control was used to define the IL-17A gate, and an isotype-matched control was used to assess non-specific binding for Foxp3. Flow cytometric data were acquired and analyzed using FCS Express software (Version 7; De Novo Software, Pasadena, CA, USA) by an investigator who was blinded to the group allocation.

### 4.8. Statistical Analysis

All data were analyzed using GraphPad Prism 8.0.2 and expressed as mean ± standard deviation (SD). Normality was assessed using the Shapiro–Wilk test, and homogeneity of variance was assessed using the Brown-Forsythe and Bartlett’s tests. When the assumption of normality was severely violated, non-parametric tests were used as the alternative analysis (the Mann–Whitney U test for two-group comparisons and the Kruskal–Wallis test followed by Dunn’s post hoc test for multiple-group comparisons). However, in the present study, the data were approximately normally distributed; therefore, given the relatively small sample sizes, parametric tests were actually applied. When the two variance tests yielded conflicting results, Brown-Forsythe was used as the primary criterion because it is more robust to non-normality and small sample sizes. For comparisons between two groups, Student’s *t*-test was used when variances were equal, and Welch’s *t*-test was used when variances were unequal. Comparisons among multiple groups were performed using one-way analysis of variance (ANOVA) followed by Tukey’s post hoc test. When Brown-Forsythe indicated that the assumption of homogeneity of variance was violated, Welch’s ANOVA followed by the Games-Howell post hoc test was used as the alternative analysis. A *p* value < 0.05 was considered statistically significant.

## Data Availability

The original contributions presented in this study are included in the article/Supplementary Materials. Further inquiries can be directed to the corresponding author.

## Supplementary Materials

The following supporting information can be downloaded at: https://www.mdpi.com/article/10.3390/ijms27219846/s1.
