# Supplementary material for: Dihydromyricetin Ameliorates Exercise-Induced Acute Hepatic Dysfunction and Inflammation in Association with the Restoration of the Th17/Treg Balance in Mice
Source: Int J Mol Sci. 2026 Nov 3;27(21):9846. doi: 10.3390/ijms27219846 (PMC13763823; doi:10.3390/ijms27219846)
Supplement: Supplementary file 1 [file ijms-27-09846-s001.zip › ijms-4587666-supplementary.pdf]

A

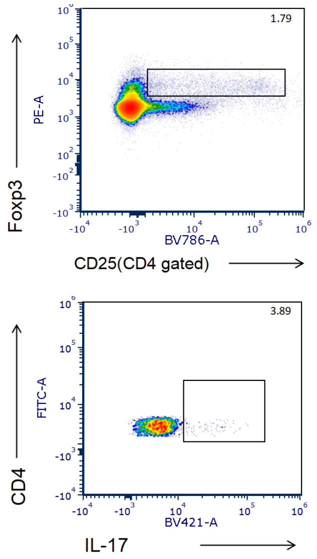

B

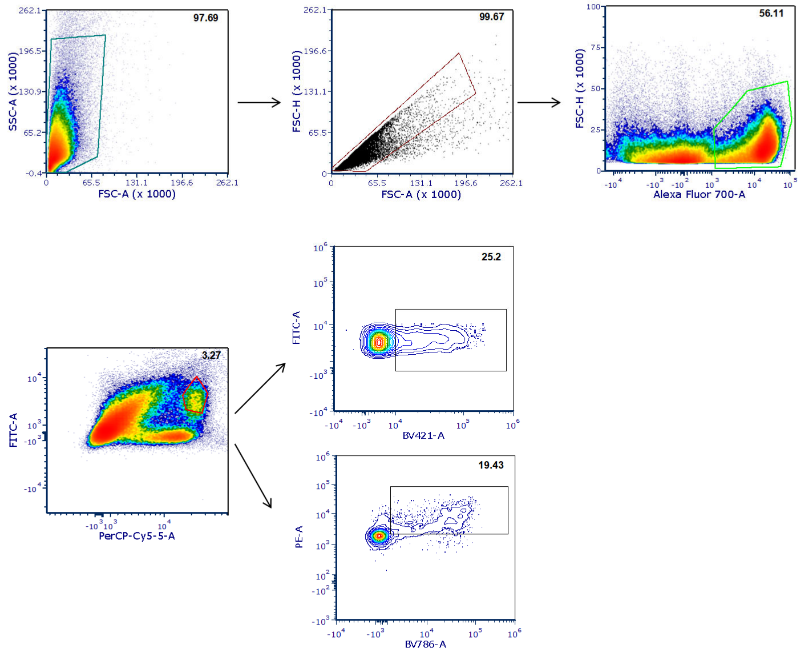

Supplementary Figure S1. Isotype/FMO controls and gating strategy for hepatic Th17 and Treg cells.

(A) Isotype control for Foxp3 (Treg, upper panel) and FMO control for IL-17A (Th17, lower panel). (B) Gating strategy for hepatic Th17 and Treg cells. Lymphocytes were first gated on FSC-A/SSC-A, followed by single cells on FSC-A/FSC-H. Debris and dead cells were excluded based on FSC/SSC morphology. CD45+ cells were gated on Comp-Alexa Fluor 700-A versus FSC-H, and CD3+CD4+ T cells on Comp-PerCP-Cy5.5-A versus Comp-FITC-A. Th17 cells (IL-17A+) were gated on Comp-BV421-A versus Comp-FITC-A, and Treg cells (CD25+Foxp3+) on Comp-BV786-A versus Comp-PE-A. The FMO control was used to define the IL-17A gate, and the isotype control was used to assess non-specific binding for Foxp3.
